# Copper(I)-binding properties of de-coppering drugs for the treatment of Wilson disease. α-Lipoic acid as a potential anti-copper agent

**DOI:** 10.1038/s41598-018-19873-2

**Published:** 2018-01-23

**Authors:** Julia Smirnova, Ekaterina Kabin, Ivar Järving, Olga Bragina, Vello Tõugu, Thomas Plitz, Peep Palumaa

**Affiliations:** 1 Department of Chemistry and Biotechnology, Tallinn University of Technology, Akadeemia tee 15, 12618 Tallinn, Estonia; 2 Wilson Therapeutics AB, Västra Kungsgatan 3, S-111 43 Stockholm, Sweden

## Abstract

Wilson disease is an autosomal recessive genetic disorder caused by loss-of-function mutations in the P-type copper ATPase, *ATP7B*, which leads to toxic accumulation of copper mainly in the liver and brain. Wilson disease is treatable, primarily by copper-chelation therapy, which promotes copper excretion. Although several de-coppering drugs are currently available, their Cu(I)-binding affinities have not been quantitatively characterized. Here we determined the Cu(I)-binding affinities of five major de-coppering drugs – D-penicillamine, trientine, 2,3-dimercapto-1-propanol, *meso*-2,3-dimercaptosuccinate and tetrathiomolybdate – by exploring their ability to extract Cu(I) ions from two Cu(I)-binding proteins, the copper chaperone for cytochrome c oxidase, Cox17, and metallothionein. We report that the Cu(I)-binding affinity of these drugs varies by four orders of magnitude and correlates positively with the number of sulfur atoms in the drug molecule and negatively with the number of atoms separating two SH groups. Based on the analysis of structure-activity relationship and determined Cu(I)-binding affinity, we hypothesize that the endogenous biologically active substance, α-lipoic acid, may be suitable for the treatment of Wilson disease. Our hypothesis is supported by cell culture experiments where α-lipoic acid protected hepatic cells from copper toxicity. These results provide a basis for elaboration of new generation drugs that may provide better therapeutic outcomes.

## Introduction

Wilson disease is characterized by loss-of-function mutations in a P-type copper ATPase, ATP7B, which is expressed mostly in liver^1,2^. The WD protein has dual roles, it is functioning in the transport of copper into the trans-Golgi network, for incorporation into the plasma protein ceruloplasmin^3,4^, and into the bile, for excretion of excess cellular copper^5^. Defective ATP7B functioning is causing reduced incorporation of copper into ceruloplasmin and copper accumulation primarily in liver and in brain, leading to liver disorders and/or neuropsychiatric symptoms^6^.

Unlike many other genetic disorders, Wilson disease is treatable, primarily by copper-chelation therapy, which promotes copper excretion. Several de-coppering drugs have been used for the treatment of Wilson disease, two of which have also been approved by the FDA. D-penicillamine (PA), the first orally administered copper-chelating agent available, was approved for therapeutic use in 1956^7^. PA induces copper excretion into urine^8^; however, it also has many adverse effects^9^. The second oral copper chelating drug, trientine (TR), was approved in 1982. TR also acts by enhancing urinary excretion of copper, however, it is better tolerated than PA^9,10^. In addition to these two major drugs, two other copper-chelating compounds have been used for treatment of Wilson disease in the past: an injectable drug, British anti-Lewisite (BAL or dimercaptopropanol), which was used in the UK in 1951^11^ and dimercaptosuccinate (DMS), which has been used in China for half a century already^12^. In Western medicine, BAL and DMS are used primarily for the treatment of arsenic, mercury, and lead poisoning^13,14^. The fifth de-coppering drug selected for our study is tetrathiomolybdate (TTM), which was introduced in 1984 and was used in a limited number of patients with Wilson disease. Initial studies with ammonium TTM^15^ and a recently completed Phase II clinical study with bis-choline TTM^16^, demonstrate that TTM acts rapidly, resulting in improvements in copper control and this is accompanied by improved neurologic outcomes and stabilization of liver function, with a favorable safety profile^17^.

De-coppering drugs should compete for copper ions with cellular Cu(I)-binding proteins, for which the Cu(I)-binding affinities are known^18^. However, the Cu(I)-binding affinities of de-coppering drugs, which are of fundamental importance for understanding their therapeutic action, are currently not known. In the current study, Cu(I)-binding affinities of de-coppering drugs were determined from their competition with two cellular Cu(I)-binding proteins, Cox17 and metallothionein (MT), which have different Cu(I)-binding affinities^18^. These proteins were reconstituted to form Cu_1_Cox17 and Cu_10_MT metalloforms and incubated with increasing concentrations of the de-coppering drugs. Demetallation of the copper proteins was monitored by electrospray ionization mass spectrometry (ESI MS)^18^ and the copper-binding affinities were determined from dose-dependent demetallation curves. The absolute values for dissociation constants (K_d_) of Cu(I)-drug complexes were obtained by comparing demetallation potencies of de-coppering drugs with those of copper chelators with known copper-binding affinities such as dithiotreitol (DTT) and diethyl dithiocarbamate (DETC). Analysis of structure-activity relationships suggested that an endogenous biologically active substance, α-lipoic acid (LA), may serve as a potential de-coppering drug. LA was able to protect hepatic cells from copper toxicity *in vitro*, which further supports its potential as an anti-copper agent.

## Methods

### Reagents

Chemical reagents: PA, TR, BAL, DMS, LA, ammonium TTM, diethylammonium DETC were purchased from Sigma-Aldrich, DTT (ultrapure) from USB Corporation, DLA from Santa Cruz Biotechnology. Bischoline TTM (WTX101) was provided by Wilson Therapeutics AB. Rabbit apo-MT2A was purchased from Bestenbalt LLC and apo-Cox17_3S-S_ was produced as described previously^19^. Recombinant human Cu,Zn-SOD was purchased from Biovision Inc. All solutions were prepared immediately before the experiments in 20 mM ammonium acetate, pH 7.3 buffer in the absence of organic solvents. To avoid oxidation of DTT and proteins, the buffer was saturated with argon.

### Reconstitution of proteins with Cu(I) ions

Lyophilized apo-Cox17 was dissolved in 20 mM ammonium acetate, pH 7.3 containing 50 µM DTT and metallated at 1 µM concentration with one equivalent of Cu(I)–DTT complex in the presence of 50 µM DTT. Cu_10_MT2 was reconstituted in the presence of 10 mM DTT from 3 µM apo-MT2 dissolved in 20 mM ammonium acetate, pH 7.3 by addition of 10-fold excess of Cu(I)–DTT. The stock solution of Cu(I)–DTT complex was prepared by dissolving Cu(II)-acetate at 1.3 mM concentration in argon-saturated 20 mM ammonium acetate containing 10 mM DTT at pH 7.3.

### ESI-MS settings and determination of copper-binding affinities

DTT is suitable for applications in ESI MS as being a nonionic compound it does not suppress the ionization efficiency of proteins during ESI-MS substantially, which enables detection of protein peaks even if ligand is present in millimolar concentrations. The Cu(I)-binding affinity of DDT has been studied extensively^18,20^, and was therefore used as a standard for determination of Cu(I)-binding affinities of DETC, PA, TR, TTM, BAL and DMS. It should be mentioned that DTT is air sensitive and its Cu(I)-binding affinity depends on pH of solution^20^. Therefore all experiments with DTT have been conducted in oxygen-free argon-saturated solutions at pH = 7.3, where the Cu(I)-binding affinity has reliably been determined (K_d_ = 5.01 × 10^−16^ M)^20^. Since DETC, PA, TR TTM and DMS are ionic compounds, they lead to ionic suppression of protein peaks in ESI MS spectra. However, ionic suppression is low at submillimolar concentrations and presumably similar for all protein peaks. In a standard experiment, the reconstituted protein was incubated with increasing concentrations of copper chelators for 1 min and samples were injected into the electrospray ion source of an Agilent Technology 6540 UHD Accurate-Mass Q-TOF MS instrument (Agilent) by a syringe pump at 7 μL/min. ESI-MS spectra were recorded for 10 min in the m/z region from 100 to 3,000 Da using the following instrument parameters: capillary voltage = 3500 V, drying gas = 4 l/min, drying gas temperature 100 °C, nebulizer 15 psig, skimmer voltage = 65 V. ESI MS spectral composition was stabilized after 5 min of monitoring time and spectra between 8–10 min were averaged for further analysis. Obtained ESI MS spectra were deconvoluted using the Mass Hunter software (Agilent). The fractional content of metallated protein forms was calculated from the integrated peak areas for metallated and nonmetallated protein forms. C_50_ values for compounds have been calculated by fitting the dose-dependent demetallation curve to the simple 1:1 binding isotherm (in case of Cu_1_Cox17) or Hill equation (in case of Cu_10_MT). K_d_ values for the Cu(I)-protein complexes at pH 7.3, 20 mM ammonium acetate, and 25 °C were determined using previously determined K_d_ values for protein-Cu(I) complexes^18^ and the re-estimated apparent K_d_ for the Cu(I)–DTT complex of 5.01 × 10^−16^ M^20^. K_d_ values for PA, TR, BAL, DMS and TTM, were determined by the ability of these ligands to extract Cu(I) ions from Cu_1_Cox17 or Cu_10_MT in comparison with DTT (in case of Cu_1_Cox17) or DETC (in case of Cu_10_MT) respectively as described above.

### Demetallation of Cu,Zn-SOD with TTM

Commercial lyophilized Cu,Zn-SOD was dissolved in argon-saturated 20 mM ammonium acetate pH 7.3 to the final concentration of 5 µM, and injected at different time points into an Agilent Technology 6540 UHD Accurate-Mass Q-TOF MS instrument by a syringe pump at 7 μL/min. ESI-MS spectra were recorded for 10 min in the m/z region from 500 to 3,000 Da using the instrument parameters presented above. ESI MS spectrum of the sample exposed two peaks: Cu,Zn-SOD and partially demetallated Zn-SOD (Fig. S9A). Following incubation of the sample with 10 µM ammonium TTM, there was a time-dependent shift in intensities of the peaks, whereas peak of Cu,Zn-SOD disappeared and peak of apo-SOD appeared in the spectrum (Fig. S9A). Spectral changes could be interpreted with extraction of Cu(I) and Zn(II) ions from Cu,Zn-SOD and Zn-SOD and formation of Cu-SOD and apo-SOD. The demetallation process occurred with a half-life of 23 ± 14 min (Fig. S9B).

### Cell culture

Huh7 hepatocyte derived cellular carcinoma cell line were cultured in Dulbecco’s Modified Eagle’s Medium (DMEM, Gibco) supplemented with 10% Fetal Bovine Serum (FBS, Gibco) and 50 U/ml penicillin, 50 µg/ml streptomycin solution (PAA) in an incubator at 37 °C and 5% CO_2_. The medium was changed every 2–3 days and cells were split using Trypsin-EDTA solution (Gibco).

### Cell viability measured by WST-1

The effects of CuCl_2_ on the cells were determined using the cell viability assay WST-1 (Roche). WST-1 assay allows colorimetric measurement of cell viability due to reduction of tetrazolium salts to water-soluble formazan by viable cells. 105 cells were seeded in triplicates in a 96 well plate and cultivated for 24 h. Various concentration (0–100 µM) of CuCl_2_ were added and the measurements were performed 24 hours after cells treatment. The experiments with no CuCl_2_ added were used as a negative control. 5 µl/well of WST-1 reagent was added to 100 µl of cell culture medium, incubated at 37 °C for 2 h and absorbance was measured at 450 nm using TECAN Genios Pro microplate reader. For LA testing, cells in triplicates were preincubated with various concentrations (0–100 µM) of LA (dissolved in ethanol) for 24 h and medium was changed to fresh medium, containing 50 µM of CuCl_2_ and various concentrations (0–100 µM) of LA. Cell viability was determined after 24 h incubation with WST-1 test. Experiments were performed twice.

## Results and Discussion

### Demetallation of Cu_1_Cox17 by the de-coppering drugs

**Figure 1:**
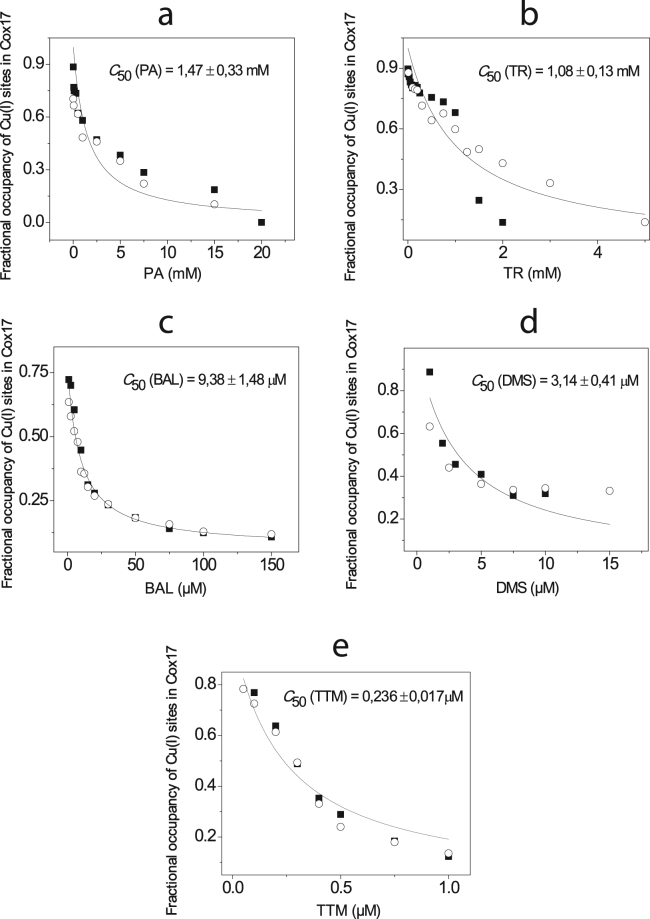
Determination of the relative Cu(I)-binding affinity of de-coppering drugs in competition with Cu_1_Cox17. Fractional occupancy of Cu(I)-binding sites in Cox17 at different concentrations of PA (**a**), TR (**b**), BAL (**c**), DMS (**d**) and TTM (**e**) in a metal competition experiment. Conditions: Cox17 1 μM; 20 mM ammonium acetate, pH = 7.3, DTT 50 μM; T = 25 °C. Results of duplicate experiments are presented with different symbols. The solid line shows the fitting curve with hyperbolic equation (y = P1*(1 − [x/(P2 + x)]) + P3), where P2 = C_50_.

All de-coppering drugs were able to demetallate Cu_1_Cox17; however, it occurred at different concentrations with different drugs. Demetallation potency of compounds was defined as the concentration where 50% of Cu_1_Cox17 was demetallated and was denoted as C_50_. PA and TR extracted Cu(I) from Cu_1_Cox17 at millimolar concentrations with the corresponding C_50_(PA) = 1.47 mM (Figs 1A and S1) and C_50_(TR) = 1.08 mM (Figs 1B and S1). BAL extracted Cu(I) from Cu_1_Cox17 at micromolar concentrations with C_50_(BAL) = 9.38 μM (Figs 1C and S3). DMS and TTM showed very low C_50_ values: C_50_(DMS) = 3.14 μM (Figs 1E and S4) and the C_50_(TTM) was less than 1 μM (Figs 1F and S5). C_50_ values were determined also for reference compounds, DTT (C_50_[DTT] = 3.10 mM) (Fig. S6) and DETC (C_50_[DETC] = 5.28 μM) (Fig. S7). Based on the linear relationship between C_50_ and K_d_ values and the known K_d_ value for DTT^20^, we calculated the K_d_ values for studied compounds and the results are presented in Table 1. As an example we present here the mathematics for calculation of K_d_ value for DETC. DETC was 587 times more effective Cu(I) chelator than DTT (C_50_[DTT] / C_50_[DETC] = 587), which is in line with earlier results obtained with a different ESI-MS setup^18^. Consequently the K_d_ for Cu(I)-DETC complex should also be 587 times lower as that for DTT and therefore K_d_(DETC) = 5.01 × 10^−16^ / 587 = 8.53 × 10^−19^ M.

**Table 1:** Determination of K_d_ values for de-coppering drugs. The efficiency of de-coppering drugs to demetallate Cu_1_Cox17 and/or Cu_10_MT (C_50_) was in linear correlation with K_d_ values for Cu(I)-binding complexes (conditions: 20 mM ammonium acetate, pH 7.3, 25 °C).

Ligand	C_50_ (mM) for Cu_1_Cox17	K_d_ (M)
DTT	3.10 ± 0.26	5.01 × 10^−16*^
DETC	0.00528 ± 0.00032	8.53 × 10^−19^
PA	1.47 ± 0.33	2.38 × 10^−16^
TR	1.08 ± 0.13	1.74 × 10^−16^
BAL	0.00938 ± 0.00148	1.52 × 10^−18^
DMS	0.00314 ± 0.00041	5.07 × 10^−19^
TTM	<0.001	<1.6 × 10^−19^
DLA	0.498 ± 0.098	8.05 × 10^−17^
**Ligand**	**C**_**50**_ **(mM) for Cu**_**10**_ **MT**	**K**_**d**_ **(M)**
DETC	0.772 ± 0.087	8.53 × 10^−19^
TTM	0.0211 ± 0.0014	2.32 × 10^−20^

*Taken from reference^20^.

### Demetallation of Cu_10_MT by the de-coppering drugs

**Figure 2:**
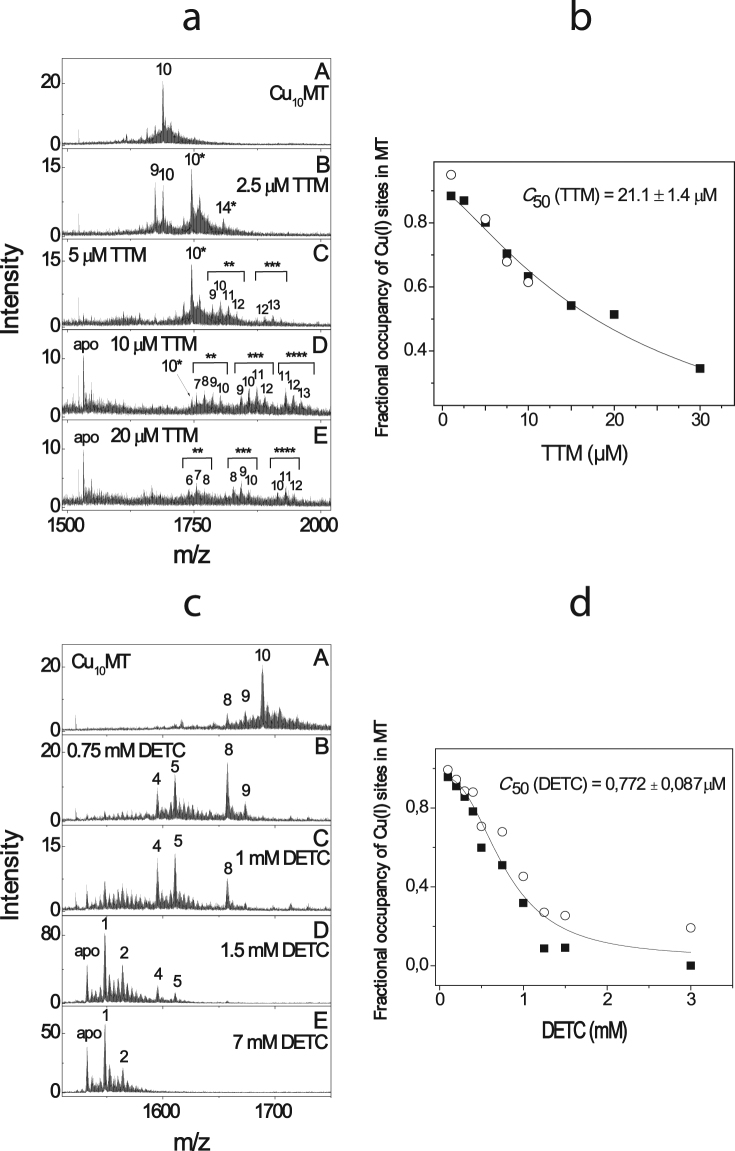
Determination of the relative Cu(I)-binding affinity of TTM and DETC in competition with Cu_10_MT. ESI-MS spectra of Cu_10_MT in the presence of 1 μM–20 μM TTM (**a**) and 0.1–7 mM DETC (**c**). Conditions: MT 3 μM; 20 mM ammonium acetate, pH = 7.3, DTT 10 mM; T = 25 °C. Ions with a charge state + 5 are shown; numbers on the peaks denote the metal stoichiometry of the complex. Number of asterisks denotes number of TTM molecules in the complex. Fractional occupancy of Cu(I)-binding sites in MT at different concentrations of TTM (**b**) and DETC (**d**) in a metal competition experiment. Results of duplicate experiments are presented with different symbols. The solid line shows the fitting curve with Hill equation (y = START + (END − START) * x^n / (K^n + x^n)), where K = C_50_.

In the second series of experiments, we explored the ability of different de-coppering drugs to extract copper from Cu_10_MT, which has 42 times higher Cu(I)-binding affinity as compared to Cu_1_Cox17^18^. TTM demetallated Cu_10_MT at micromolar concentrations through a stepwise process. First, a complex Cu_10_MT-TTM was formed with 1:1 stoichiometry (Fig. 2A). Multiple peaks appeared at higher concentrations of TTM, indicative of the binding of a second, third and fourth TTM molecule to the complex and the simultaneous build-up of apo-MT (Fig. 2A). The binding of multiple TTM ions appeared to lead to the opening of Cu(I)-thiolate clusters and dissociation of Cu(I) ions from the protein. As dissociation of Cu(I)-thiolate clusters of Cu_10_MT occurred cooperatively we did not observe hyperbolic demetallation curves like in case of Cu_1_Cox17. By this reason fitting of the Cu_10_MT demetallation curves by the influence of TTM was performed by using the Hill equation^18^. A C_50_(TTM) value of 21.1 μM was obtained by taking into account all CuMT-TTM complexes (Fig. 2B). Both salts of TTM, ammonium and bis-choline TTM, behaved similarly in ESI-MS experiments. Earlier HPLC/ICP experiments have also demonstrated the binding of TTM to CuMT^21^; however, the exact composition of ternary complexes remained unclear.

DETC also extracted Cu(I) from Cu_10_MT (Fig. 2C) at millimolar concentrations and the corresponding C_50_(DETC) was equal to 0.77 mM (Fig. 2D). Other de-coppering drugs were unable to demetallate Cu_10_MT up to millimolar concentrations. Based on the linear relationship between C_50_ and K_d_ values and the known K_d_ value for DETC (see Table 1), the K_d_ value for TTM was calculated (K_d_ = 2.32 × 10^−20^ M) and inserted into Table 1. The absolute value of K_d_ for Cu_10_MT of 4.1 × 10^−16^ M was first determined in 2010^18^ based on the absolute value of available K_d_ for the Cu(I)-DTT complex known at that time. Subsequently, the K_d_ for the Cu(I)-DTT complex has been corrected from 7.94 × 10^−12^ M^22^ to 5.01 × 10^−16^ M (pH 7.3)^20^. Based on the corrected absolute dissociation constant for Cu(I)-DTT complex, the corrected K_d_ for Cu_10_MT was calculated to be 2.59 × 10^−20^ M. TTM had almost equal copper-binding affinity (K_d_ = 2.32 × 10^−20^ M) to that of MT. Indeed, TTM can form a 1:1 TTM-Cu_10_MT complex at concentrations equimolar to MT, whereas at higher concentrations (from 5 to 30 μM) it can extract Cu(I) ions from Cu_10_MT (Fig. 2A).

### Clinical implications of demetallation by de-coppering drugs

The obtained information can be applied to the clinical context. For a sustainable treatment effect in Wilson disease, de-coppering drugs must extract copper from intracellular copper stores, which have, however, different Cu(I)-binding affinities. MT has a high affinity for Cu(I) ions and only one de-coppering drug studied, TTM, had the ability to demetallate Cu_10_MT at low micromolar concentrations. For estimation of the physiological concentration of TTM, we used data from oncology patients where TTM 180 mg/day resulted in C_max_ of 5.7 µM^23^. At such concentrations, TTM can partially demetallate CuMT, which most probably forms the basis of its fast and efficient therapeutic action.

MT has one of the highest copper-binding affinities among cellular Cu(I) proteins^18^ and TTM may therefore extract copper also from several other essential copper proteins. Other cellular copper chaperones and copper transporters have copper-binding affinities similar to that of Cox17^18^ and most probably they can also be demetallated by TTM. Intracellular copper enzymes Cu,Zn-SOD (antioxidative defense) and mitochondrial cytochrome c oxidase (mitochondrial electron transfer) have copper-binding affinities comparable to that of MT^18^. However, the dissociation of metal ions from the active sites of these enzymes is very slow^18^, which might protect them from fast demetallation by TTM. It has been shown that TTM does not inhibit mitochondrial cytochrome c oxidase activity in hepatic mitochondrial preparations at concentrations up to 100 µM even after 16-h incubation^24^, strongly suggesting that TTM cannot demetallate cytochrome c oxidase. Nevertheless, in the same study, TTM inhibited Cu,Zn-SOD in a time-dependent manner^24^. Our data confirm that 10 µM TTM can extract copper from 5 µM Cu,Zn-SOD with a half-life of 23 min (Fig. S8). Thus, treatment with high doses of TTM may demetallate Cu,Zn-SOD, which should be taken into account in refinement of therapeutic doses for TTM.

The K_d_ values for PA and TR were 2.38 × 10^−16^ M and 1.74 × 10^−16^ M, respectively. Therapeutic doses of PA and TR (750–1500 mg/day^17^) are substantially higher than that for TTM. The current study suggests that PA and TR cannot effectively compete with CuMT at therapeutic concentrations (approximately 50–100 µM); however, they can partially compete with copper chaperones. At therapeutic concentrations, BAL and DMS effectively demetallated copper chaperones and only partially CuMT; however, the action of DMS is restricted mainly to the extracellular space since it is unable to cross biological membranes.

### Structure-affinity relationship analysis and identification of new copper-binding drugs

**Figure 3:**
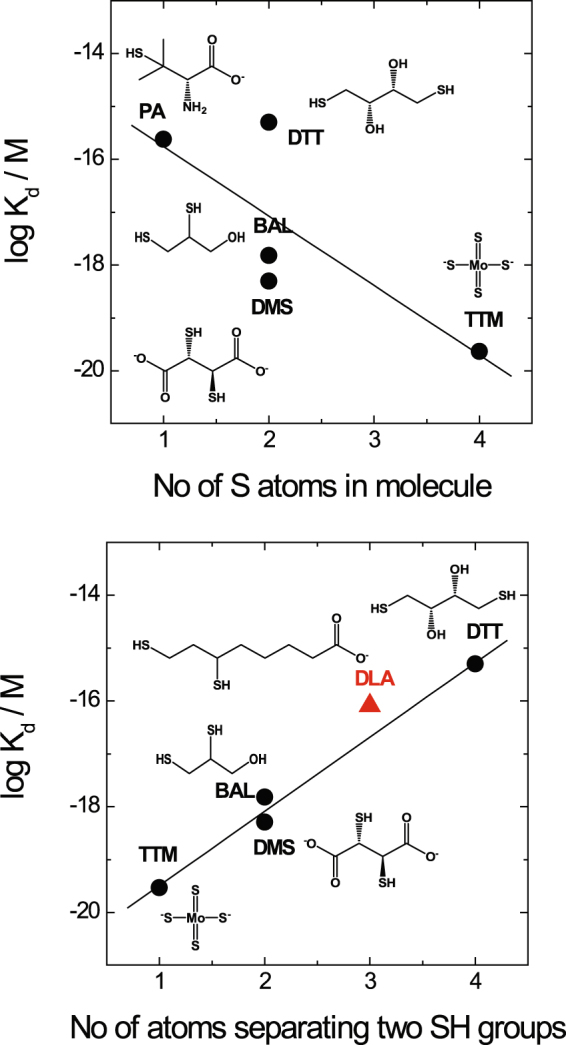
Structure-activity relationships. Correlation between the number of sulfur atoms in the molecule of copper chelators and their Cu(I)-binding affinity (**a**); correlation between the number of carbon atoms separating two SH groups in the molecule of copper chelators including DLA and their Cu(I)-binding affinity (**b**).

The Cu(I)-binding affinity of the de-coppering drugs studied varied by four orders of magnitude depending upon the molecular structure of the compounds. One can speculate that the number of sulfur-containing groups in the molecule, which are well adapted for the coordination of Cu(I) ions, is an important determinant of the binding affinity. Indeed, a positive correlation between the Cu(I)-binding affinities and the number of the S-atoms in the molecule (Fig. 3A) was observed: TTM, with four S-atoms, has the highest Cu(I)-binding affinity, and PA with only one S-atom has the lowest affinity. However, the Cu(I)-binding affinity of DTT is considerably different from that of BAL and DMS, although all these compounds carry two SH groups. Thus, not only the number, but also the position of the sulphur-containing groups appears to affect the Cu(I)-binding affinity of compounds. Indeed, the Cu(I)-binding affinity decreased substantially with increasing number of atoms between the sulfhydryl groups: TTM > BAL, DMS > DTT (Fig. 3B). This structure-affinity relationship explains why TTM has the highest Cu(I) affinity of all de-coppering agents studied and opens new perspectives for the rational design of new copper-binding drugs.

In order to test the validity of this structure-activity relationship and its applicability for design of new copper-binding drugs, we looked for biomolecules with three intercalating atoms between two SH groups and found that dihydrolipoic acid (DLA) fits these criteria. The experimental Cu(I)-binding affinity of DLA was equal to K_d_ = 8.05 × 10^−17^ M (Fig. S9, Table 1), which fits well the predicted relationship between logK_d_ and the number of atoms between two SH groups, thus confirming the structure-activity relationship. The Cu(I)-binding affinity of DLA was 2 and 3 times higher than that of TR and PA respectively, however, the affinity was not high enough to directly decopper intracellular high-affinity Cu stores like Cu_10_MT when tested experimentally (data not shown). *In vivo*, DLA is formed from α-lipoic acid (LA) by reduction in a cellular environment. LA is a well-tolerated food supplement that is able to cross biological membranes. Considering its higher Cu(I)-binding affinity than that of chelators approved for the treatment of Wilson disease (PA, TR), good membrane and blood brain barrier permeability^25^ as well as very good tolerability^26^, we suggest that LA might be a suitable de-coppering drug for treatment of Wilson disease. Some exploratory attempts to use LA in Wilson disease have been reported^27,28^, however, the potency of LA in the treatment of Wilson disease has not been explored in cellular experiments.

### Effect of LA on cellular copper toxicity

**Figure 4:**
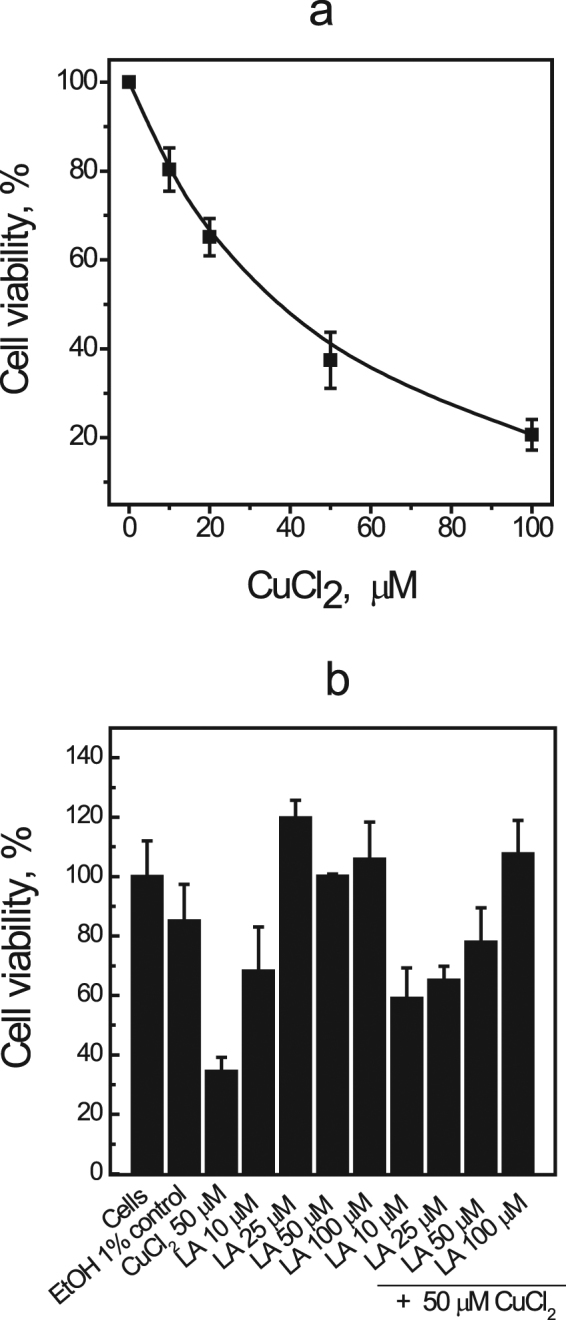
Effect of LA on cellular copper toxicity. Viability of Huh7 cells determined by WST-1 assay after 24 h of exposure to CuCl_2_ (**a**). Viability of Huh7 cells after 24 h incubation with different concentrations of LA and additional treatment with 50 µM CuCl_2_ for next 24 h, measured with WST-1 test. Data are shown as mean ± SD.

To further explore the potential of LA as a de-coppering drug we performed preliminary experiments with Huh7 hepatic cell lines to test the putative protective effect of LA against copper toxicity. Similar experimental setup has been used earlier to demonstrate the protective effect of PA on copper toxicity exerted on hepatic cell lines^29,30^. The results in Fig. 4 demonstrate that copper is toxic to Huh7 cells with LD_50_ of approximately 30 μM of CuCl_2_ (Fig. 4a) and LA exerts a clear dose-dependent protective effect on toxicity of 50 μM CuCl_2_ (Fig. 4b). The protective effect on cellular level supports the potential therapeutic role of LA in treatment of Wilson disease, however, the full spectrum of therapeutic activities of LA should be evaluated in further cellular and animal studies.

## Electronic supplementary material

Supplementary Information
