# Supplementary material for: Copper(I)-binding properties of de-coppering drugs for the treatment of Wilson disease. α-Lipoic acid as a potential anti-copper agent
Source: Sci Rep. 2018 Jan 23;8:1463. doi: 10.1038/s41598-018-19873-2 (PMC5780470; doi:10.1038/s41598-018-19873-2)
Supplement: Supplementary file 1 — Supplementary Information [file 41598_2018_19873_MOESM1_ESM.pdf]

Copper(I)-binding properties of de-coppering drugs for the treatment of Wilson disease

Julia Smirnova[†], Ekaterina Kabin[†], Ivar Järving[†], Olga Bragina[†], Vello Tõugu[†], Thomas Plitz[‡] and Peep Palumaa[†,*]

[†]Department of Chemistry and Biotechnology, Tallinn University of Technology, Akadeemia tee 15, 12618 Tallinn, Estonia

[‡]Wilson Therapeutics AB, Västra Kungsgatan 3, S-111 43 Stockholm, Sweden

[*] Corresponding author: E-mail: peep.palumaa@ttu.ee, Tel +372 5025559, Fax: +372 6204401

SUPPLEMENTARY INFORMATION

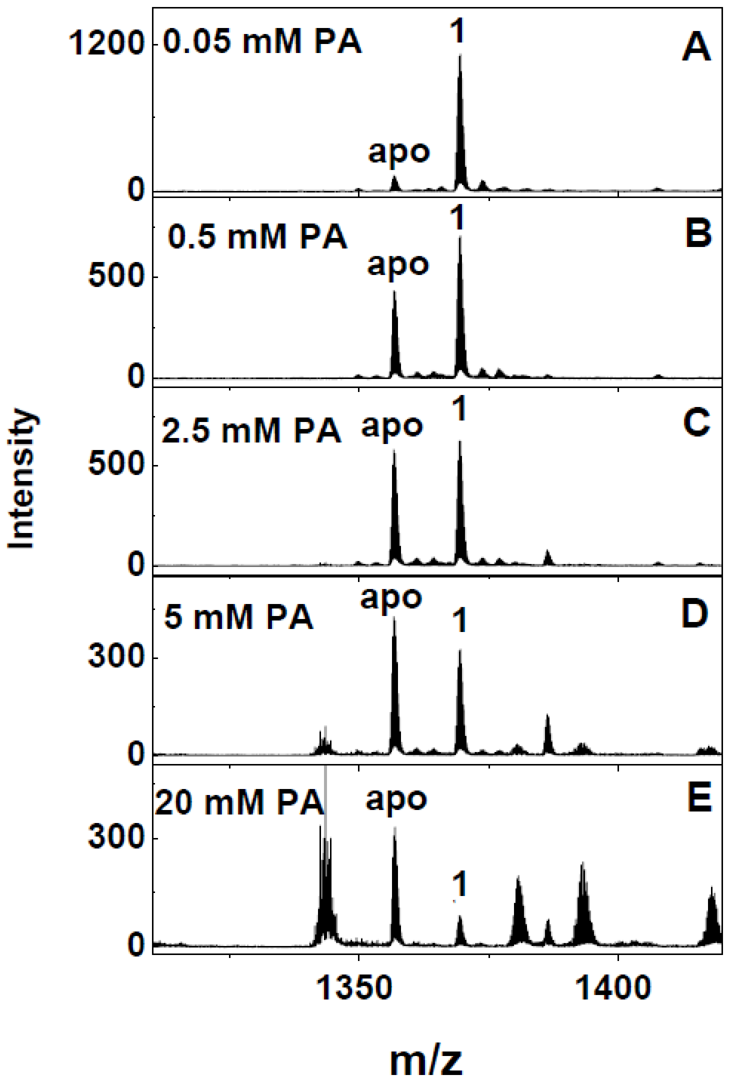

Fig. S1. Determination of the relative Cu(I)-binding affinity of PA in competition with Cu_1Cox17.

ESI-MS spectra of Cu_1Cox17 in the presence of 1 μM – 20 mM PA. Conditions: Cox17 1 μM; 20 mM ammonium acetate, pH=7.3, DTT 50 μM; T=25°C. Ions with a charge state +5 are shown; numbers on the peaks denote the metal stoichiometry of the complex.

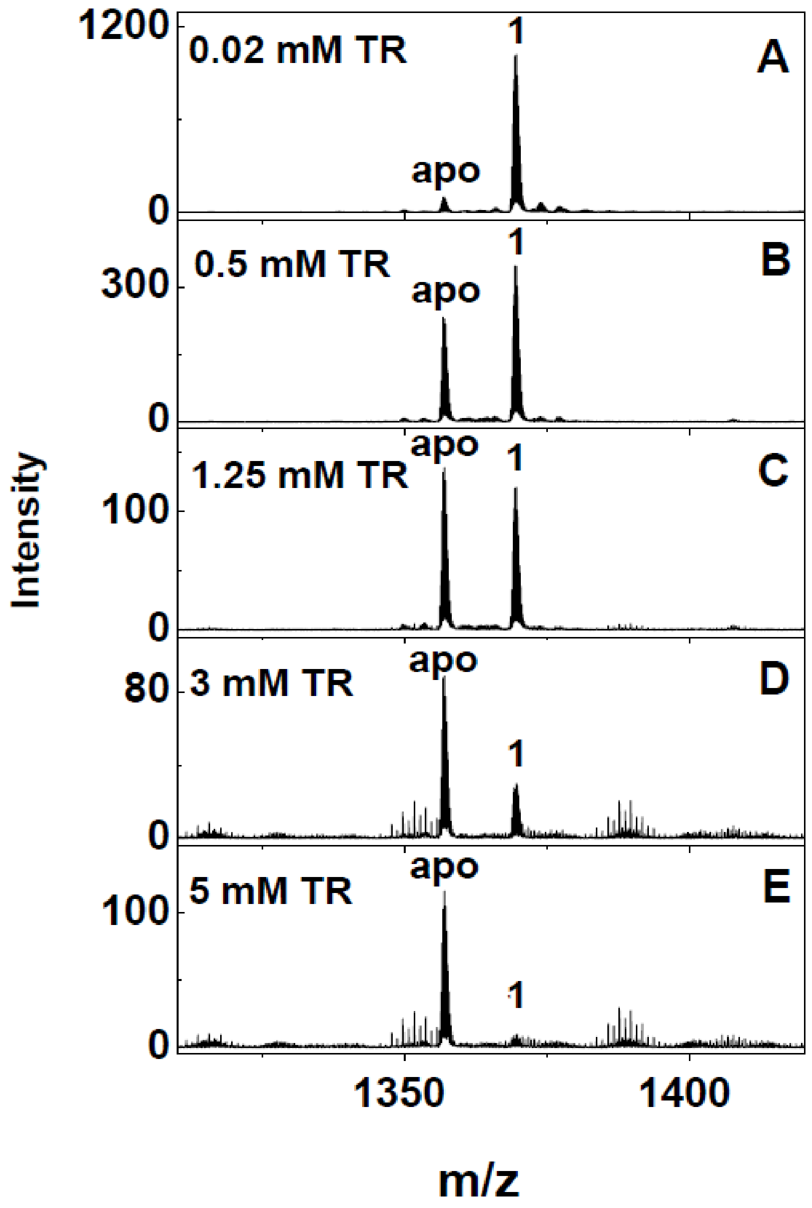

Fig. S2. Determination of the relative Cu(I)-binding affinity of TR in competition with Cu_1Cox17.

ESI-MS spectra of Cu1Cox17 in the presence of 1 µM – 5 mM TR. Conditions: Cox17 1 µM; 20 mM ammonium acetate, pH=7.3, DTT 50 µM; T=25°C. Ions with a charge state +5 are shown; numbers on the peaks denote the metal stoichiometry of the complex.

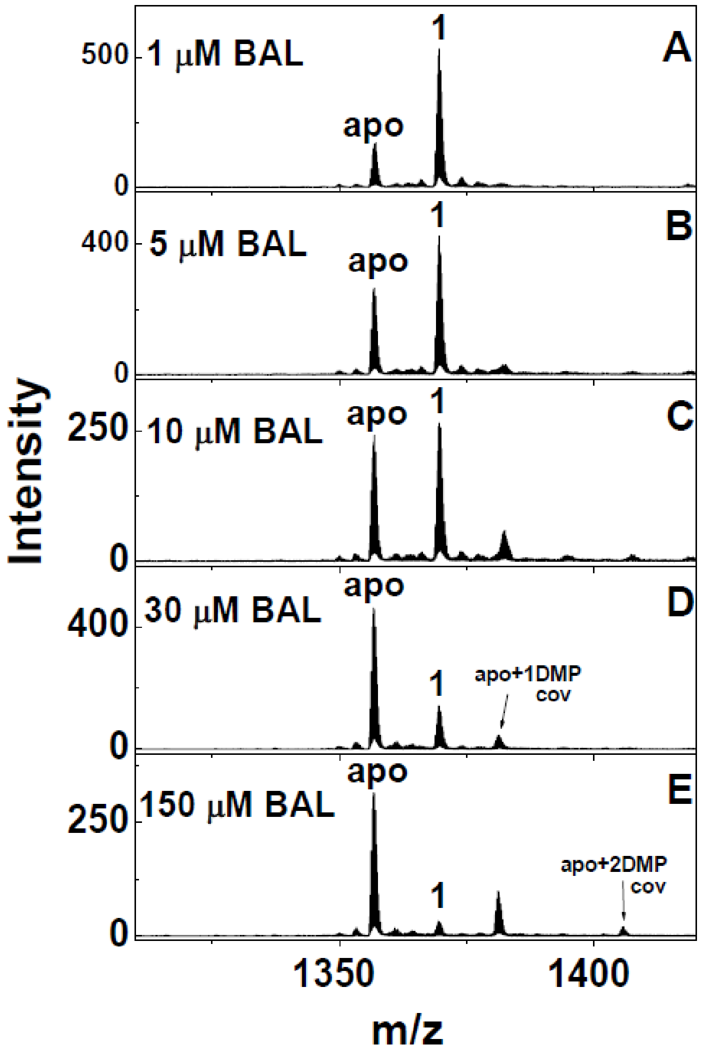

Fig. S3. Determination of the relative Cu(I)-binding affinity of BAL in competition with Cu1Cox17.

ESI-MS spectra of Cu_1Cox17 in the presence of 1 – 150 μM BAL. Conditions: Cox17 1 μM; 20 mM ammonium acetate, pH=7.3, DTT 50 μM; T=25°C. Ions with a charge state +5 are shown; numbers on the peaks denote the metal stoichiometry of the complex.

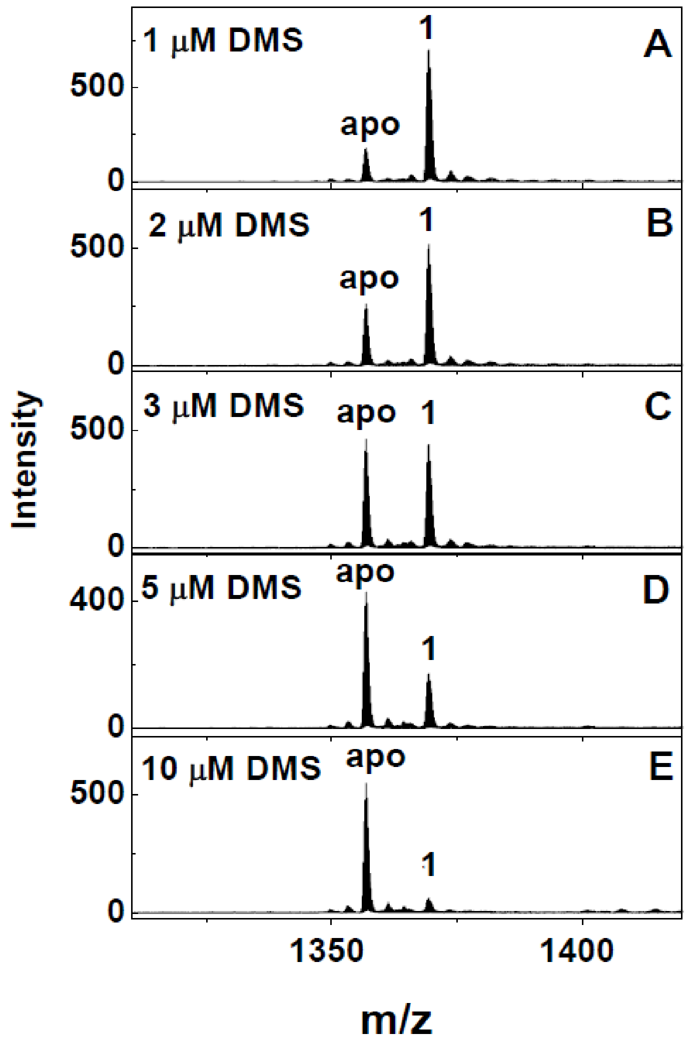

Fig. S4. Determination of the relative Cu(I)-binding affinity of DMS in competition with Cu_1Cox17.

ESI-MS spectra of Cu_1Cox17 in the presence of 1 μM – 15 μM DMS. Conditions: Cox17 1 μM; 20 mM ammonium acetate, pH=7.3, DTT 50 μM; T=25°C. Ions with a charge state +5 are shown; numbers on the peaks denote the metal stoichiometry of the complex.

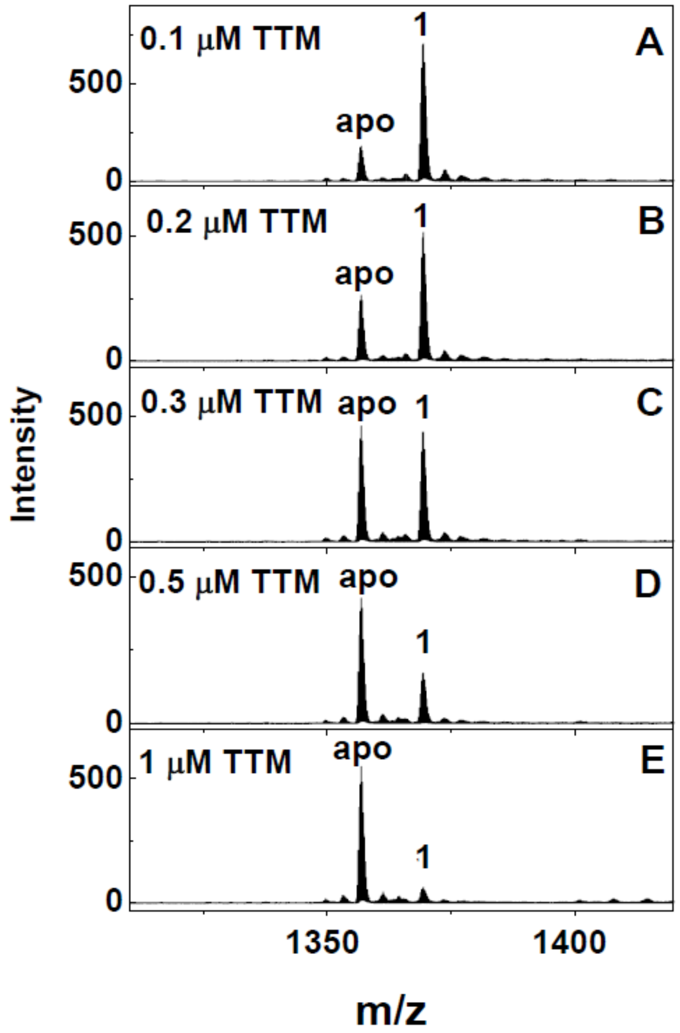

Fig. S5. Determination of the relative Cu(I)-binding affinity of ammonium TTM and bis-choline TTM in competition with Cu_1Cox17.

ESI-MS spectra of Cu_1Cox17 in the presence of 0.1 – 2.5 μM ammonium TTM. Conditions: Cox17 1 μM; 20 mM ammonium acetate, pH=7.3, DTT 50 μM; T=25°C. Ions with a charge state +5 are shown; numbers on the peaks denote the metal stoichiometry of the complex.

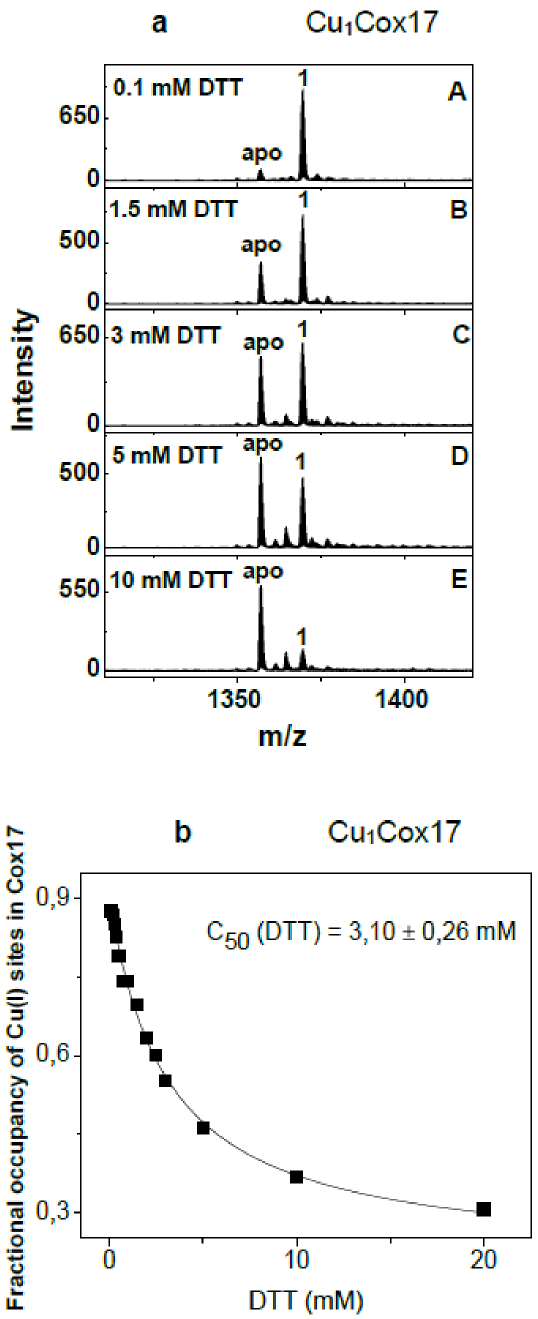

Fig. S6. Determination of the relative Cu(I)-binding affinity of DTT in competition with Cu_1Cox17.

(*a*) ESI-MS spectra of Cu_1Cox17 in the presence of 0.1 – 10 mM DTT. Conditions: Cox17 1 µM; 20 mM ammonium acetate, pH=7.3, DTT 50 µM; T=25°C. Ions with a charge state +5 are shown; numbers on the peaks denote the metal stoichiometry of the complex. (*b*) Fractional occupancy of Cu(I)-binding sites in Cox17 at different concentrations of DTT in a metal competition experiment. The solid line shows the fitting curve with hyperbolic equation (y=P1*[1-(x/[P2+x])]+P3), where P2 = C_{50}.

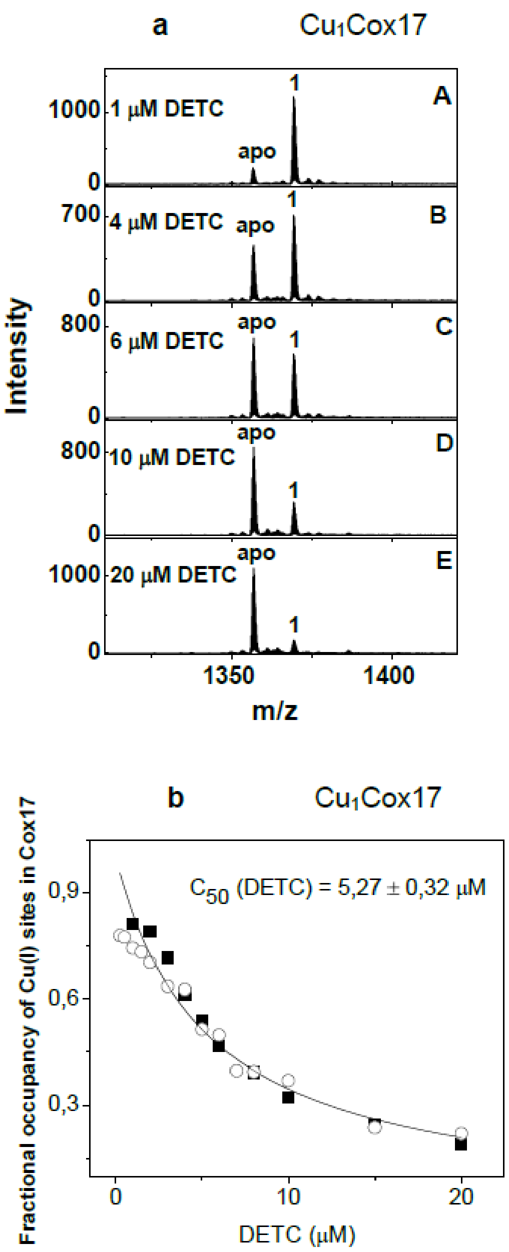

Fig. S7. Determination of the relative Cu(I)-binding affinity of DETC in competition with Cu1Cox17.

(a) ESI-MS spectra of Cu_1Cox17 in the presence of 1 – 20 μM DETC. Conditions: Cox17 1 μM; 20 mM ammonium acetate, pH=7.3, DTT 50 μM; T=25°C. Ions with a charge state +5 are shown; numbers on the peaks denote the metal stoichiometry of the complex. (*b*) Fractional occupancy of Cu(I)-binding sites in Cox17 at different concentrations of DETC in a metal competition experiment. Results of duplicate experiments are presented with different symbols. The solid line shows the fitting curve with hyperbolic equation (y= P1*[1-(x/[P2+x])]+P3), where P2 = C_{50}.

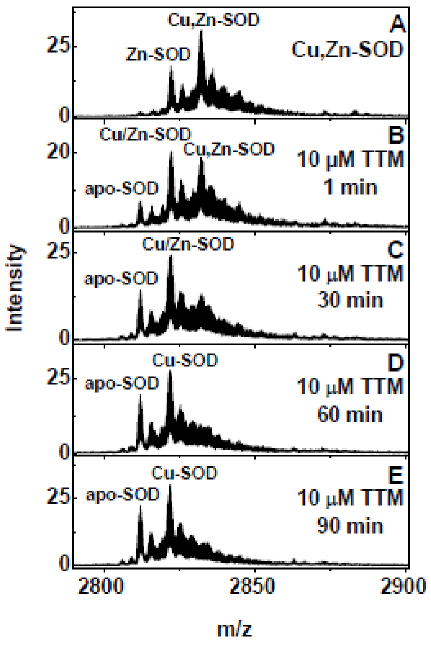

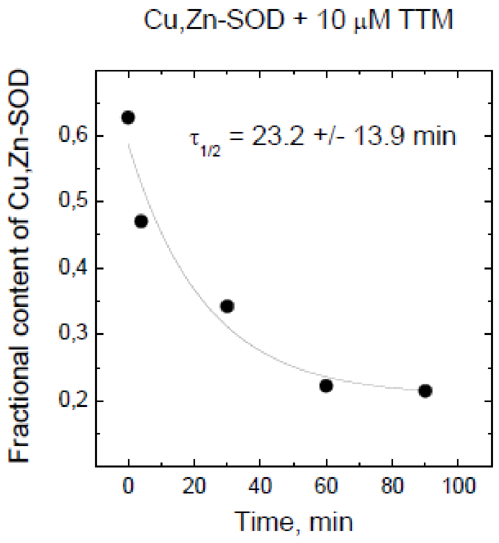

Fig. S8. Dissociation of Cu(I) ions from Cu,Zn-SOD by the influence of TTM.

(*a*) ESI-MS spectra of 5 µM Cu,Zn-SOD 2a in the presence of 10 µM TTM. Conditions: 20 mM ammonium acetate, pH=7.3, DTT 10 mM; T=25°C. Ions with a charge state +6 are shown; (*b*) Demetallation kinetics of 5 µM Cu,Zn-SOD by the influence of 10 µM TTM.

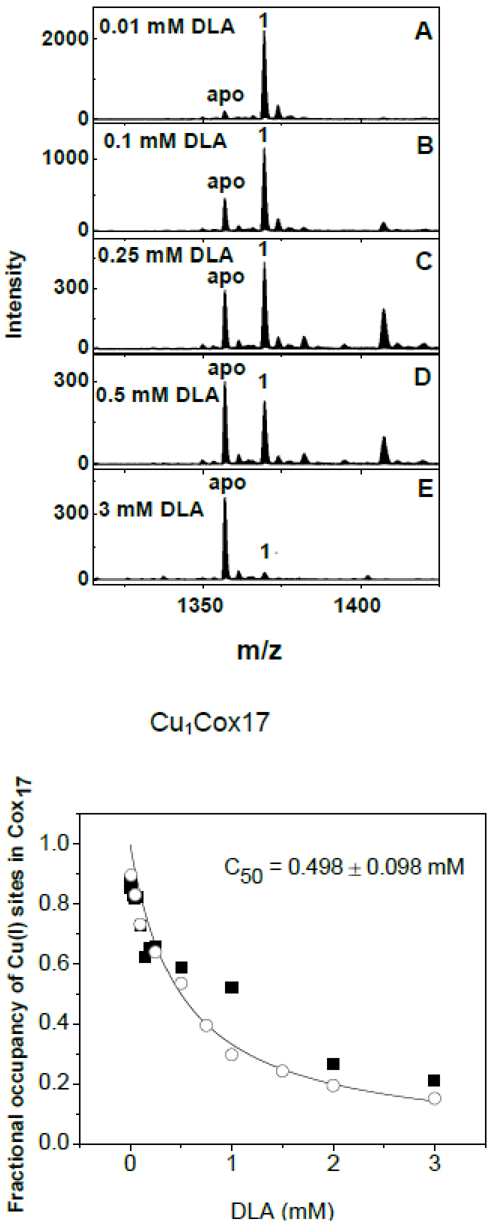

Fig. S9. Determination of the relative Cu(I)-binding affinity of DLA in competition with Cu_1Cox17.

(*a*) ESI-MS spectra of Cu_1Cox17 in the presence of 1 μM – 3 mM DLA. Conditions: Cox17 1 μM; 20 mM ammonium acetate, pH=7.3, DTT 50 μM; T=25°C. Ions with a charge state +5 are shown; numbers on the peaks denote the metal stoichiometry of the complex. (*b*) Fractional occupancy of Cu(I)-binding sites in Cox17 at different concentrations of DMS in a metal competition experiment. Results of duplicate experiments are presented with different symbols. The solid line shows the fitting curve with hyperbolic equation (y= P1*[1-(x/[P2+x])]+P3), where P2 = C_{50}.
